# Comprehensive Analysis of Cellular Galectin-3 Reveals No Consistent Oncogenic Function in Pancreatic Cancer Cells

**DOI:** 10.1371/journal.pone.0020859

**Published:** 2011-06-16

**Authors:** Alexander Hann, Anja Gruner, Ying Chen, Thomas M. Gress, Malte Buchholz

**Affiliations:** Department of Gastroenterology, Endocrinology and Metabolism, Philipps-University of Marburg, Marburg, Germany; Stanford University School of Medicine, United States of America

## Abstract

Galectin-3 (Gal-3), a 31 kDa member of the family of beta-galactoside-binding proteins, has been implicated in the progression of different human cancers. However, the proposed roles differ widely, ranging from tumor-promoting cellular functions and negative impact on patient prognosis to tumor-suppressive properties and positive prognostic impact. We and others have previously identified Gal-3 as overexpressed in pancreatic cancer as compared to chronic pancreatitis and normal pancreatic tissue. The purpose of this study was thus the comprehensive analysis of putative cellular functions of Gal-3 by transient as well as stable silencing or overexpression of Gal-3 in a panel of 6 well-established pancreatic cancer cell lines. Our results confirm that galectin-3 is upregulated at the mRNA level in pancreatic cancer and strongly expressed in the majority of pancreatic cancer cell lines. In individual cell lines, transient knockdown of Gal-3 expression resulted in moderate inhibitory effects on proliferation, migration or anchorage-independent growth of the cells, but these effects were not consistent across the spectrum of analyzed cell lines. Moreover, functional effects of the modulation of Gal-3 expression were not observed in stable knockdown or overexpression approaches *in vitro* and did not alter the growth characteristics of nude mouse xenograft tumors *in vivo*. Our data thus do not support a direct functional role of Gal-3 in the malignant transformation of pancreatic epithelial cells, although paracrine or systemic effects of Gal-3 expression are not excluded.

## Introduction

Pancreatic ductal adenocarcinoma, the most common form of pancreatic cancer, has an overall 5-year survival rate of <5%. Over 80% of patients present at an advanced disease stage without the option for potential curative tumor resection [1], [2]. Although chemotherapy combined with a small-molecule inhibitor targeting EGF receptor signaling has recently been shown to result in a modest, but significant increase in survival in locally advanced or metastatic tumors, the prognosis remains dismal, with median survival not exceeding 6.2 months [3]. Thus, new diagnostic and therapeutic approaches that improve outcome are urgently required.

The family of beta-galactoside-binding proteins (Galectins) consists of 14 members in mammals. A common feature that distinguishes Galectins from other lectins is their carbohydrate-recognition domain. Galectin-3 (Gal-3), the 31 kDa member of this family, has been linked to a variety of tumors, albeit with widely differing roles [4]. Gal-3 overexpression in cancerous tissue has been associated with a poor prognosis in different cancer types including hepatocellular carcinoma [5], [6], clear cell renal carcinoma [7], [8] and bladder cancer [9]. Conversely, reduced Gal-3 expression in tumor tissue compared with normal tissue was reported in ovarian cancer [10], uterine adenocarcinoma [11], breast cancer [12], [13] and cervical neoplasia [14]. In colorectal cancer, reports have been contradictory. Some authors describe a positive association of high levels of Gal-3 expression with advanced tumor stages and poor prognosis [15]–[17], whereas others correlate decreased expression of Gal-3 with poor prognosis [18]–[20]. In gastric cancer, Okada et al reported that reduced Gal-3 expression correlated with lymph node metastasis and advanced tumor stage [21], whereas two other groups identified increased expression of Gal-3 in gastric cancer, but did not find a correlation with histopathological differentiation or tumor progression [22], [23].

Despite the conflicting results concerning a possible prognostic role of Gal-3 expression, some reports have described tumor-promoting functions of Gal-3 in tumor cell lines *in vitro*. Knockdown of Gal-3 resulted in reduced cell migration and cell growth in prostate cancer cells [24], enhanced apoptosis induction in gastric cancer cells [25], and inhibited *in vitro* colony formation as well as nude mouse xenograft induction in breast cancer cells [26]. We and others have previously shown that Gal-3 is consistently overexpressed in pancreatic cancer as compared to both chronic pancreatitis and normal pancreas [27]–[30]. However, investigations into a possible functional role of Gal-3 expression in pancreatic cancer cells have not been reported. The purpose of this study was thus to experimentally evaluate the effects of overexpression or knockdown of Gal-3 in a comprehensive set of pancreatic cancer cell lines (PaTu 8988s, PaTu 8988t, S2-007, S2-028, IMIM-PC-1 and MIA PaCa-2). Functional analyses included assays for cell viability, apoptosis, proliferation, migration and anchorage independent growth as well as tumor growth in a xenograft mouse model. Apart from isolated effects in single cell lines, modulation of Gal-3 expression had no consistent effect on tumor-relevant characteristics of pancreatic cancer cells.

## Materials and Methods

### Human tissues and cell lines

The human pancreatic adenocarcinoma cell line IMIM-PC-1 [31] was kindly provided by F.X. Real (Insitute Municipale de Investigacion Medica, Barcelona, Spain). S2-028 and S2-007 [32] were from T. Iwamura (Miyazaki Medical College, Miyazaki, Japan). MIA PaCa-2 was obtained from the American Type Culture Collection (ATCC, RMD, USA). PaTu 8988t and PaTu 8988s were kindly provided by H.P. Elsässer (Institut für Klinische Zytobiologie und Zytopathologie, Philipps Universität, Marburg, Germany). All cell lines were maintained in Dulbecco's modified minimal essential medium (GIBCO, Invitrogen Corp., NY, USA) supplemented with 10% FCS (GIBCO, Invitrogen Corp., NY, USA) and Gentamicin 0.045 mg/ml (GIBCO, Invitrogen Corp., NY, USA).

### Ethics Statement

Surgically resected pancreatic adenocarcinoma and chronic pancreatitis tissues were provided by the surgery departments at the Universities of Ulm and Homburg/Saar. Normal pancreas samples were obtained from healthy areas at the borders of chronic pancreatitis resectates. Written informed consent was obtained from all patients prior to using tissue samples. The study was approved by the ethics committee at the University of Ulm, Germany (Ethikkommission der Universitaet Ulm) as well as the ethics committee at the University of Homburg/Saar, Germany (Ethikkommission der Universitaet Homburg).

### Transfection of cell lines

Small interfering RNA (siRNA) was transfected into PaTu 8988s, S2-007 and S2-028 cells using siLentFect Lipid Reagent (Bio-Rad, Munich, Germany) according to the manufacturer's protocol. SiRNA transfection into MIA PaCa-2 cells was performed using Transmessenger reagent (Qiagen, Hilden, Germany) and IMIM-PC-1 cells were transfected with X-tremeGENE siRNA Transfection Reagent (Roche, Mannheim, Germany) according to the manufacturers' protocols, respectively. The Gal-3-specific siRNAs were: siGal-3-1, Hs_LGALS3_1 FlexiTube siRNA SI00470036 and siGal-3-2, Hs_LGALS3_2 FlexiTube siRNA SI00470043 (Qiagen). Silencer Negative Control from Ambion was used as non-silencing control.

The Gal-3 expression vector was constructed by cloning the PCR-amplified Gal-3 open reading frame into the pcDNA V3.2/V5 dest vector using the Gateway recombination cloning technology (Invitrogen Life Technologies, Karlsruhe, Germany). Following transfection of PaTu 8988t cells using Lipofectamine 2000 Transfection Reagent (Invitrogen), selection of stably transfected cell clones was performed by adding 800 µg/ml G418 to the culture medium.

A Gal-3-specific shRNA expression construct in the pGIPZ vector was purchased from Open Biosystems, Huntsville, AL, USA (cat. # RHS4430-99137619). Non-silencing shRNAmir (cat. # RHS4348, Open Biosystems) was used as the negative control. Stable transfection of the S2-007 cells was performed using Lipofectamine 2000 (Invitrogen). Stably transfected clones were selected by adding hygromycin (400 µg/ml) to the culture medium.

### RNA Extraction and qRT-PCR

RNA from cell lines was extracted using peqGold Total RNA Kit (PEQLAB Biotechnologie GmbH, Erlangen, Germany) according the manufacturer's protocol. Bulk tumor tissue samples were homogenized on dry ice/liquid nitrogen using a mortar and pestle. RNA was extracted using the RNeasy Mini Kit (Qiagen) according the manufacturer's protocol. First-strand cDNA was synthesized from 1 µg total RNA using the Omniscript RT Kit (Qiagen) according to manufacturer's protocol. Quantitative real time PCR (qRT-PCR) was performed using SYBR Green MasterMix (Applied Biosystems, Wellesley, MA, USA) and specific primer pairs designed with the PrimerExpresss program (Applied Biosystems). The following primer pairs were used for qRT–PCR: ribosomal protein, large, P0 (RPLP0) fwd: 5′-TGGGCAAGAACACCATGATG-3′; rev. 5′-AGTTTCTCCAGAGCTGGGTTGT-3′; Galectin-3 fwd: 5′-AGAGGGAATGATGTTGCCTTCC-3′; rev: ACAATGACTCTCCTGTTGTTCTCATT-3′.

### Subcellular fractionation and immunoblotting

Subcellular fractionation was performed as described previously [33]. Briefly, cells were washed twice with icecold PBS and collected by centrifugation at 1.600 r.p.m. at 4°C. Lysates were then resuspended in buffer A (10 mM Hepes pH 7.9; 10 mM KCl; 0,1 mM EDTA; 0.1 mM EGTA; 1 mM DTT; proteinase inhibitors) for 15 min and subsequently centrifuged for 20 min at 3.600 r.p.m. Supernatants were transferred to new cups and centrifuged at 14,000 r.p.m. for additional 4 min. Pellets were resuspended in 30–100 ml buffer C (20 mM Hepes pH 7.9; 0.4 M NaCl; 1 mM EDTA; 1 mM EGTA; 1 mM DTT; proteinase inhibitors) and incubated on ice for 30 min. A final centrifugation step at 14,000 r.p.m. for 10 min was performed to separate nuclear proteins from cellular debris. For Western blotting, the resulting nuclear protein extracts were electrophoresed through a 7.5% SDS–polyacrylamide gel and transferred onto PVDF Immobilon-P membranes (Millipore, Billerica, MA, USA) as described previously [34]. Membranes were probed with either anti-Gal-3 (mouse monoclonal, cat. # A3A12, abcam, Cambridge, UK), anti-ORC2 (polyclonal rabbit, cat. # 559266, BD Biosciences), anti-PARP (rabbit polyclonal, cat. # 9542, Cell Signaling Technology, Boston, MA, USA), or anti-β-actin (mouse monoclonal, cat. # A1978, Sigma-Aldrich, Saint Louis, MI, USA) antibodies, washed in TBS washing buffer and incubated with peroxidase-conjugated secondary antibodies. Enhanced chemiluminescence reaction system (Roche) was used for visualization.

### MTT cell viability assay

Cells were reseeded 24 hours after transfection into 3.9 cm^2^ dishes at 60,000 to 100,000 cells/well. After additional 24 h or 48 h of culture, medium was replaced by MTT-containing medium (thiazolyl blue, Carl Roth, Karlsruhe, Germany) and dishes were incubated for 2 h at 37°C. The MTT containing medium was replaced by solubilisation solution (10% Triton X-100 (Carl Roth), 0.1 molar hydrochloric acid (Fisher Scientific, Schwerte, Germany) dissolved in isopropanol (Sigma-Aldrich)) and extinction measured at 570 nm. 48 h values were divided by 24 h values to correct for possible variations in numbers of seeded cells.

### BrdU cell proliferation assay

DNA replication as a direct measure of mitotic activity was measured using the Cell Proliferation ELISA, BrdU chemiluminescence Kit (Sigma) according to the manufacturer's protocol. Briefly, 5,000 to 10,000 cells were reseeded 24 hours after transfection into a 96 well μClear plates (Greiner Bio-One, Frickenhausen, Germany). BrdU containing medium was added for 4 or 6 h. After removal of the BrdU containing medium, cells were fixed for 1 h and subsequently incubated with the peroxidase-conjugated anti-BrdU antibody for 1.5 h. The chemiluminescence reaction was measured in relative light units per second (rlu/s).

### Trail induced apoptosis

In order to extrinsically induce apoptosis in PaTu 8988s cells, 300,000 cells were seeded in 9.6 cm^2^ culture dishes and treated with Trail protein (R&D Systems, Minneapolis, MN) at a dosage of 25 ng/ml for 24 h.

### Cell migration assay

Modified Boyden chamber assays were performed by reseeding 20,000 to 40,000 cells resuspended in serum free media into transwell inserts with a pore size of 8 µm (BD Biosciences, Heidelberg, Germany). Inserts were suspended in 24well plates filled with medium supplemented with 1% FCS. Cells were allowed to migrate towards the lower face of the membrane for 2 or 4 h at 37°C. Non-migrated cells were removed from the inside of the inserts using cotton swabs. Migrated cells were stained by submerging the membranes in 0.2% crystal violet dissolved in 20% methanol for 10 min. Migrated cells were counted using light microscopy at a magnification of 10×.

### Soft agar assays

In order to assess the potential for anchorage-independent growth, soft agar assays were performed as described previously [35]. In brief, 1×10^4^ cells per 9.6 cm^2^ cell culture dish were seeded in DMEM/0.33% bacto-agar onto a bottom layer of DMEM/0.5% bacto-agar. Anchorage-independent growth was measured after 7 days of incubation by counting the number of viable colonies.

### Nude mouse xenografts

NMRI-*nu/nu* mice were propagated and maintained in a pathogen-free environment. Female 6–8-week-old mice were used in the experiments. To generate xenografts, 10^6^ tumor cells in 0.1 ml of serum-free DMEM were injected subcuntaneously into the flanks of the mice. Three weeks after inoculation, the mice were sacrificed, tumors were explanted and tumor sizes were determined. One half of each tumor was stored in 2% formaldehyde and embedded in paraffin for histological and immunohistochemical examination. The other half was snap frozen in liquid nitrogen for RNA and protein isolation.

## Results

### Overexpression of Gal-3 in pancreatic cancer

**Figure 1:**
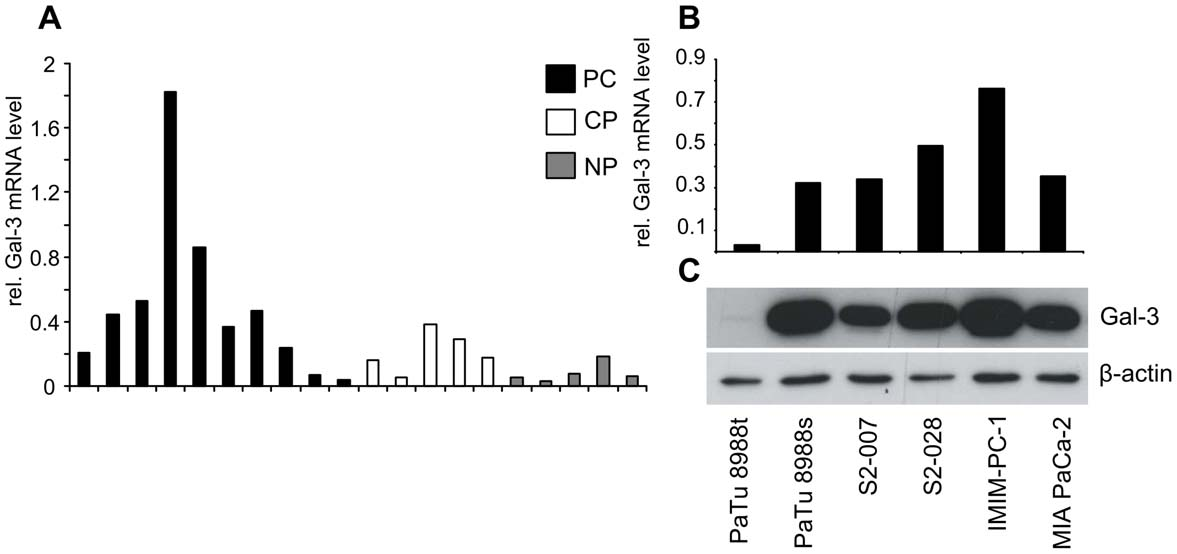
Overexpression of Gal-3 in pancreatic cancer primary tissues and cell lines. Gal-3 mRNA levels were determined by quantitative real-time PCR (qRT-PCR) (A, B). Ribosomal protein, large, P0 (RPLP0) was used as the reference gene. PC: pancreatic cancer ; CP: chronic pancreatitis ; NP: normal pancreas. Protein expression in different pancreatic cancer cell lines was determined by Western blot (**C**) analyses. β-actin was used as loading control.

In previous high-content microarray analyses, we have identified Gal-3 as one of the genes overexpressed in microdissected pancreatic cancer tissues [28]. In order to validate these results, we performed quantitative RealTime PCR (qRT-PCR) on a set of tissue samples including 10 pancreatic cancer, 5 chronic pancreatitis and 5 normal pancreatic tissue specimens. Gal-3 mRNA levels were slightly elevated in the chronic pancreatits samples, and were strongly upregulated in the majority of cancer samples (Fig. 1A), confirming the microarray data. Subsequent analysis of pancreatic cancer cell lines demonstrated strong expression, both on the mRNA as well as on the protein level, in 5 out of 6 tested cell lines (Fig. 1B).

### Expression level of Gal-3 has no influence on tumor cell growth *in vitro*

**Figure 2:**
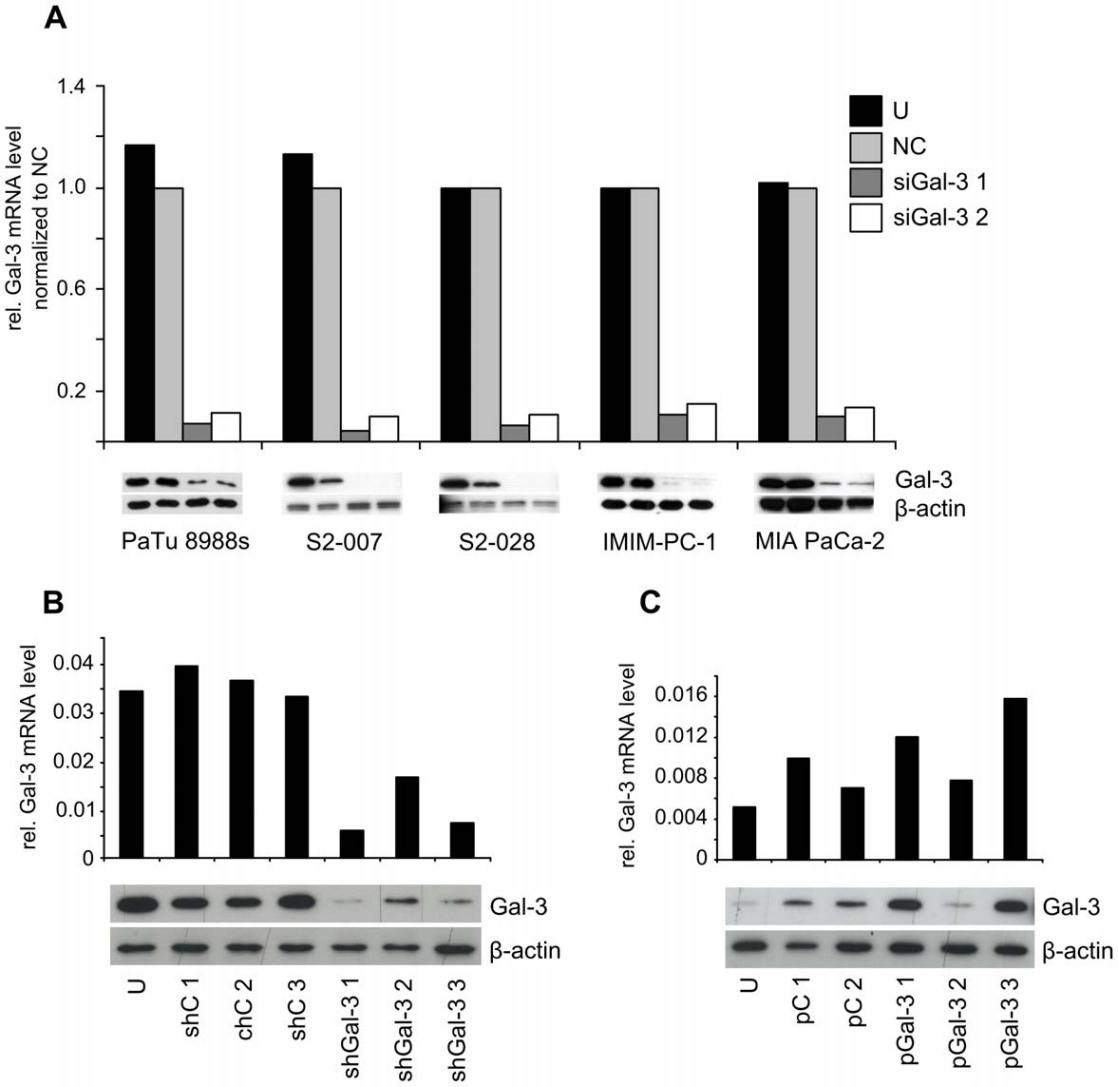
Silencing and overexpression of Gal-3 in pancreatic cancer cell lines. (**A**) Transient knockdown of Gal-3 was performed by transient transfection of multiple cell lines using two different Gal-3 specific siRNA sequences (siGal-3 1 and 2) or a non-silencing control siRNA (NC). (**B**) Stable knockdown in S2-007 was performed using shRNA expression constructs. Three Gal-3 specific shRNA transfected clones (shGal-3 1, 2 and 3) and three nonsilencing control shRNA transfected clones (shC 1, 2 and 3) were chosen for further analysis. (**C**) Stable overexpression of Gal-3 was achieved by transfection of PaTu 8988t cells with a Gal-3 expression vector (pGal-3 1, 2 and 3) or a control vector (pC 1 and 2). U denotes untransfected cells. Total cell lysates were analyzed by qRT-PCR (upper panels) and Western blot (lower panels) for Gal-3 expression levels. Gal-3 mRNA levels were determined relative to RPLP0. siGal-3 1 and 2 were normalized to NC. β-actin was used as loading control for the Western blots.

To assess the functional role of the aberrantly expressed Gal-3 in pancreatic cancer cells, Gal-3 was transiently or stably overexpressed or silenced in different pancreatic cancer cell lines and the effects analyzed in various *in vitro* functional assays. For transient silencing, two independent siRNA sequences as well as a non-silencing control siRNA were used. For each of the five cell lines analyzed (PaTu 8988s, S2-007, S2-028, IMIM-PC-1 and MIA PaCa-2), transfection reagents and –conditions were optimized to achieve knockdown efficiencies >80% (Fig. 2A).

Stable knockdown clones were generated using S2-007 cells transfected with shRNA sequences cloned in the pGIPZ vector. Three stably transfected clones as well as three control vector transfected clones were chosen for further analysis (Fig. 2B).

Since PaTu 8988t cells showed very low endogenous Gal-3 levels, this cell line was chosen for the construction of stable Gal-3 overexpressing clones. Three independent clones were established. Two of these showed moderate overexpression of recombinant Gal-3 on the mRNA level, which was much more pronounced on the protein level (Fig. 2C, lanes 4, 6). The remaining clone did not show appreciable expression of recombinant Gal-3 (Fig. 2C, lane 5), but was included in the further experiments as additional control clone. Interestingly, control clones transfected with empty vector also showed somewhat elevated Gal-3 levels (Fig. 2C, lanes 2, 3).

**Figure 3:**
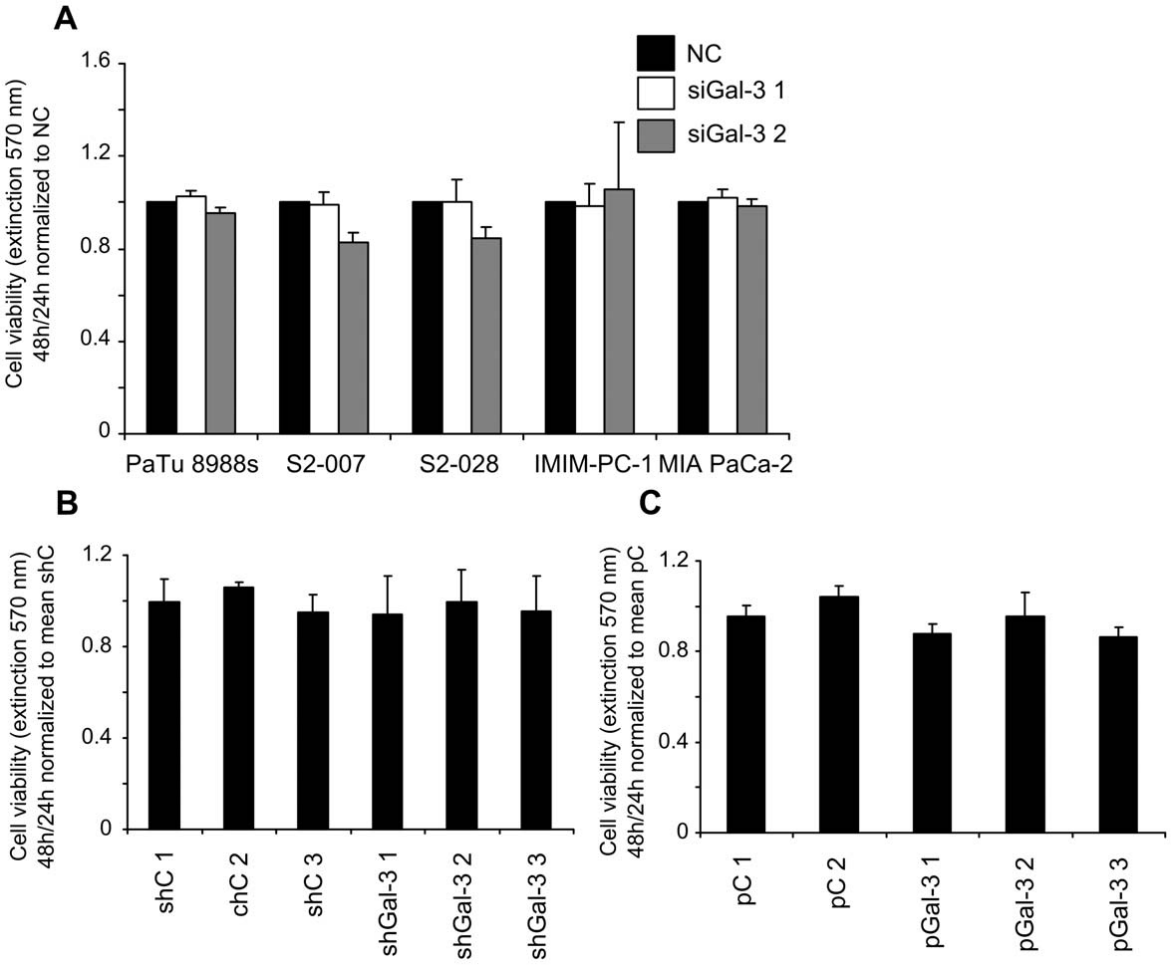
No effect of Gal-3 knockdown or overexpression on pancreatic cancer cell viability in MTT assay. Transiently Gal-3 silenced cell lines (**A**), stably Gal-3 silenced S2-007 cell clones (**B**) or stably Gal-3 overexpressing PaTu 8988t cell clones (**C**) were cultured for 24 and 48 h followed by incubation with MTT reagent. 48 h values were divided by 24 h values (to correct for possible variations in numbers of seeded cells) and normalized to control siRNA transfected cells (NC). Values of stable Gal-3 knockdown cell clones (shGal-3 1, 2 and 3) and stable Gal-3 overexpressing cell clones (pGal-3 1, 2 and 3) were normalized to the mean of control vector transfected stable cell clones (shC 1, 2, 3 and pC 1, 2) respectively. Data includes a minimum of three independent experiments.

In a first functional assay, the influence of Gal-3 expression on cell viability was analyzed by MTT assays. Neither transient knockdown in any of the 5 tested cell lines, nor stable knockdown in S2-007 cells or stable overexpression in PaTu 8988t cells had any significant effect on cell viability under normal culture conditions as compared to the suitable controls (Fig. 3 A–C).

**Figure 4:**
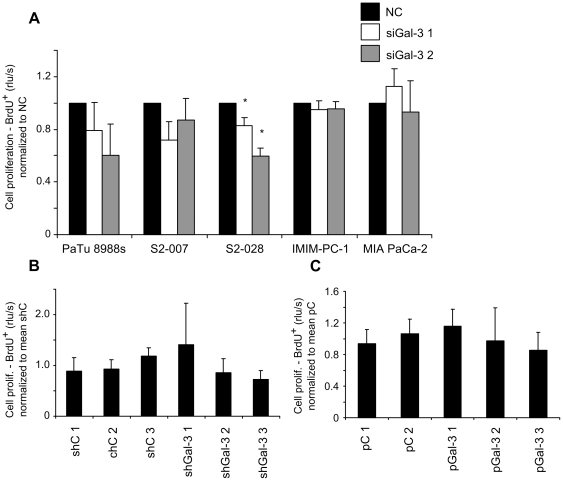
Inconsistent effect of Gal-3 on cell proliferation as measured by BrdU uptake. Transiently Gal-3 silenced cell lines (**A**), stably Gal-3 silenced S2-007 cell clones (**B**) or stably Gal-3 overexpressing PaTu 8988t cell clones (**C**) were cultured with BrdU agent for 2 or 4 h. BrdU incorporation by proliferating cells was measured by ELISA. Values of cells transiently transfected with different Gal-3 specific siRNAs (siGal-3 1 and 2) were normalized to control siRNA transfected cells (NC). Values of stable Gal-3 knockdown cell clones (shGal-3 1, 2 and 3) and stable Gal-3 overexpressing cell clones (pGal-3 1, 2 and 3) were normalized to the mean of control vector transfected stable cell clones (shC 1, 2, 3 and pC 1, 2) respectively. Data includes a minimum of three independent experiments. * Indicates *p*<0.05 as compared with control siRNA transfected cells (double-sided unpaired *t*-test).

Analysis of cell proliferation using BrdU assays revealed a moderate, but significant inhibition of proliferative activity in S2-028 cells after knockdown of Gal-3 (Fig. 4A, panel 3). However, the trend to reduced proliferation did not reach significance in PaTu 8988s and S2-007 cells, was absent in IMIM-PC-1 and even reversed in MIA PaCa-2 cells (Fig. 4A). Neither stable knockdown in S2-007 cells, nor stable overexpression of Gal-3 in PaTu 8988t cells had any significant effect on proliferation of the resulting cell clones.

**Figure 5:**
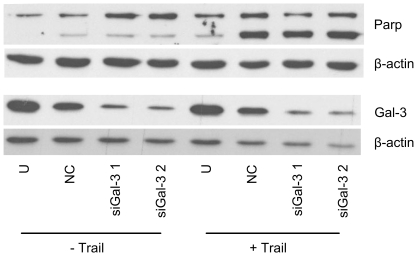
No effect of transient Gal-3 knockdown on caspase-dependent apoptosis in PaTu 8988s cells. Cells transiently transfected with Gal-3 specific siRNAs (siGal-3 1 and 2), nonsilencing control siRNA (NC) or untransfected cells (U) were analyzed by Western blot for poly ADP-ribose polymerase (PARP) cleavage. Increased cleavage, indicated by the enhanced signal of the lower band, was seen in all transfected cells. PARP cleavage was enhanced by treatment with the extrinsic apoptosis inducer Trail, but was not influenced by Gal-3 knockdown. β-actin was used as loading control. Results shown are representative of three individual experiments.

Likewise, transient Gal-3 knockdown had no effect on apoptotic activity (as measured by PARP cleavage) of PaTu 8988s cells in the absence (Fig. 5, lanes 1–4) or presence (Fig. 5, lanes 5–8) of the extrinsic apoptosis inducer Trail. PARP cleavage was induced by the transfection procedure and was more pronounced in the presence of Trail (Fig. 5, lanes 2 and 6), but was not further enhanced by knockdown of Gal-3 (Fig. 5, lanes 3–4 and 7–8).

Replication of this set of experiments under serum-free conditions produced similar results, failing to demonstrate any consistent influence of the modulation of Gal-3 expression on cell growth or apoptosis in the tested cell lines (data not shown).

### Inconsistent effect of Gal-3 on cell migration

**Figure 6:**
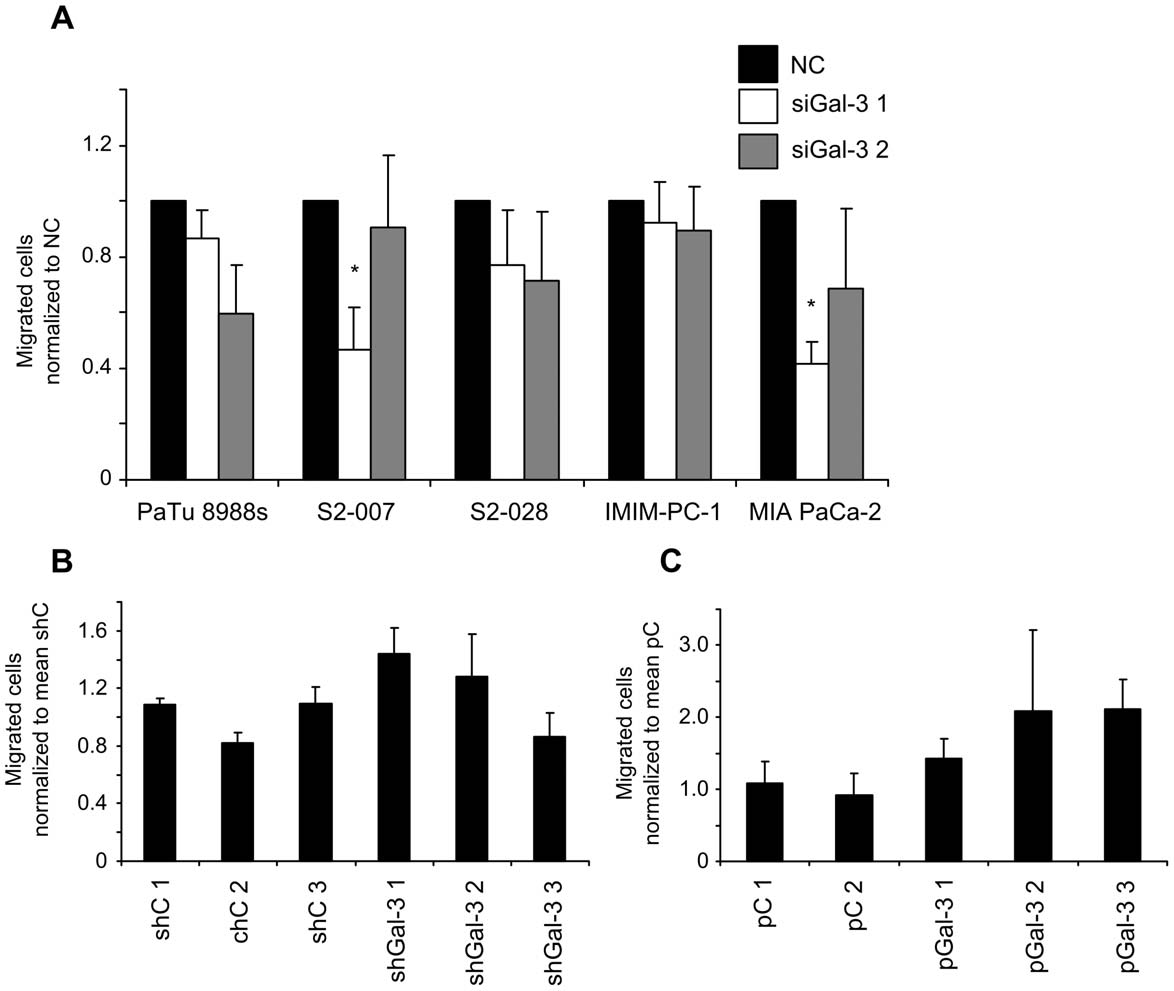
Inconsistent effect of Gal-3 knockdown and overexpression on cell migration as measured by Boyden-chamber assay. Transiently Gal-3 silenced cell lines (**A**), stably Gal-3 silenced S2-007 cell clones (**B**) or stably Gal-3 overexpressing PaTu 8988t cell clones (**C**) were cultured in Boyden-chamber inserts for 2 or 4 h. Cells migrating through the pores of the inserts along a fetal calf serum gradient were fixed, stained methylene blue and counted using light microscopy. Numbers of migrated cells transiently transfected with different Gal-3 specific siRNA (siGal-3 1 and 2) were normalized to control siRNA transfected cells (NC). Migrated stable Gal-3 knockdown cells (shGal-3 1, 2 and 3) and stable Gal-3 overexpressing cells (pGal-3 1, 2 and 3) were normalized to the mean of control vector transfected stable cell clones (shC 1, 2, 3 and pC 1, 2) respectively. Data includes a minimum of three independent experiments. * Indicates *p*<0.05 as compared with control siRNA transfected cells (double-sided unpaired *t*-test).

We ext analyzed the influence of Gal-3 expression on cell migration in a Boyden chamber assay. Cells were able to transmigrate through a filter along a fetal calf serum gradient. Numbers of transiently Gal-3 silenced migrated cells were normalized to non-silencing control siRNA transfected cells. Transient knockdown of Gal-3 resulted in a trend towards decreased cell migration in three out of five analysed cell lines, although this effect did not reach significance in most instances (Fig. 6A). Moreover, this effect was not reproduced in the S2-007 cell clones with stable Gal-3 knockdown (Fig. 6, B). In reverse analogy to the results of the transient knockdown experiments, stable overexpression of Gal-3 in PaTu 8988t cells induced a trend towards increased cell migration (Fig. 6, C), although this trend again did not reach significance and was also apparent in clone pGal-3 2, which showed low or absent expression of recombinant Gal-3 (see Fig. 2C). Taken together, modulation of Gal-3 expression had a mild and rather inconsistent effect in the Boyden chamber assays, thus arguing against an important role of cellular Gal-3 in directed migration of pancreatic cancer cells.

### Inhibition of Gal-3 expression impairs anchorage independent growth in a subset of pancreatic cancer cell lines but has no effect on growth of xenograft tumors *in vivo*

**Figure 7:**
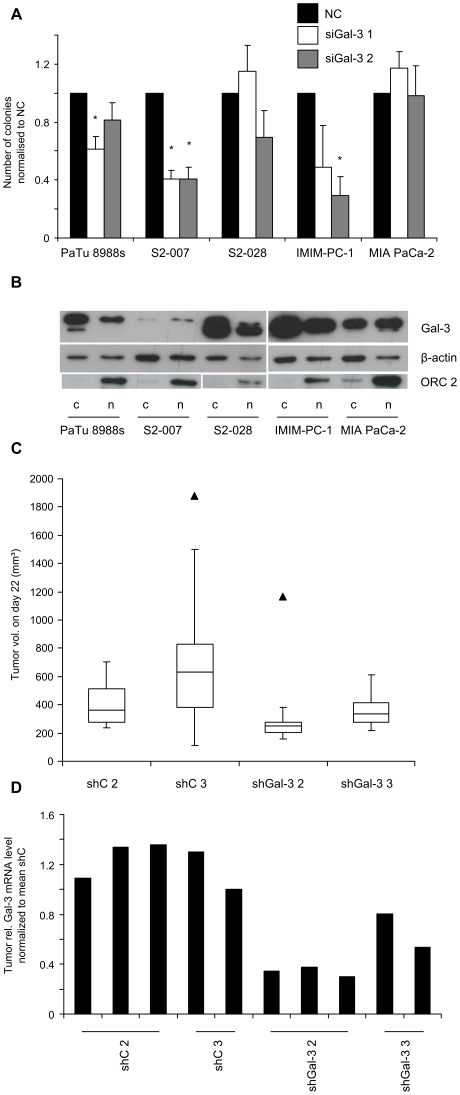
Gal-3 knockdown decreases anchorage independent cell growth, but has no effect on tumor growth in a xenograft mouse model. (**A**) Gal-3 was transiently silenced in pancreatic cancer cell lines using two independent siRNA sequences (siGal-3 1 and 2). Knockdown and control cells were seeded in soft agar and colony formation assessed after 7 days. Results were normalized to control siRNA transfected cells (NC). Data includes a minimum of three independent experiments. * Indicates p<0.05 as compared with control siRNA transfected cells (double-sided unpaired *t*-test). (**B**) Western blot analysis of nuclear (n) and cytoplasmatic (c) protein fractions derived from pancreatic cancer cell lines. Gal-3 is shown in the upper lane. β-actin staining was used as loading control. Staining for the nuclear factor ORC2 was used to assess the purity of the subcellular fractions. (**C**) Xenograft tumors were induced in nude mice by subcutaneous injection of two stable Gal-3 knockdown S2-007 cell clones (shGal-3 2 and 3) and two nonsilencing control shRNA transfected S2-007 clones (shC 2 and 3). Six mice per experimental group were analyzed. Box-and-whisker plots illustrate tumor volumes on day 22 post injection. Whiskers denote 1.5× interquartile range (IQR). Values outside this range are shown as filled triangles. (**D**) Gal-3 mRNA levels in bulk tissue of xenograft tumors where determined by qRT-PCR. RPLP0 was used as the reference gene.

The potential for anchorage-independent growth of the cancer cells was assessed by evaluating the numbers of colonies formed in soft agar assays. Transient knockdown of Gal-3 resulted in a clear reduction in colony formation capacity for the cell lines PaTu 8988s, S2-007 and IMIM-PC-1, with the strongest effect and highest statistical significance observed for S2-007 cells (Fig. 7A). Conversely, S2-028 and MIA PaCa-2 cells were not affected by the Gal-3 knockdown. Since previous reports had indicated that tumor-promoting functions of Gal-3 in other cell systems may depend on the subcellular localization of the protein [26], [36], [37], we analyzed whether the differential effect of Gal-3 knockdown colony forming capacity correlated with different subcellular localization of Gal-3 in the tested cell lines. Gal-3 protein was evenly distributed between nucleus and cytosol in MIA PaCa-2 and S2-007 cells and was slightly overrepresented in the cytosolic fractions in PaTu 8988s, S2-028 and IMIM-PC-1 cells (Fig. 7B), thus showing no correlation with sensitivity towards Gal-3 knockdown.

We then tested if the effect on anchorage-independent growth in S2-007 cells also translated into differences in tumor growth *in vivo*.

To this end, xenograft tumors were induced in nude mice by subcutaneous injection of S2-007 cells with stable Gal-3 knockdown or control vector-transfected cells, respectively. No significant difference in tumor volumes between Gal-3 knockdown and control tumors was observed (Fig. 7C). Histological examination of the tumors also did not reveal any systematic differences in differentiation, local invasiveness or microvessel density between the different groups (data not shown).

In order to confirm persistent reduction of Gal-3 levels in the knockdown tumors, mRNA was prepared from bulk tumor tissue and analyzed by qRT PCR. As expected, Gal-3 mRNA levels were significantly lower in the tumors derived from the S2-007 knockdown clones than in those derived from the control vector-transfected clones (Fig. 7D).

## Discussion

Deregulation of Gal-3 expression has been described in several human cancers, although the prognostic significance of the observed changes remains a matter of controversy [37], [38]. Pancreatic ductal adenocarcinoma is among those human tumors in which a significant overexpression of Gal-3 both on mRNA as well as on the protein level is well established [27], [30]. Our own results from microarray analyses of microdissected pancreatic tumor tissues indicated that Gal-3 is expressed by the tumor cells themselves rather than by the stromal cells which typically make up the bulk of the tumor [28]. In the current study, we confirm overexpression of Gal-3 mRNA in the tumor tissues by qRT-PCR and demonstrate that strong Gal-3 expression is retained in the majority of pancreatic cancer cell lines *in vitro*.

As is the case with data on the prognostic role in cancer, reports on putative cellular functions of Gal-3 in cancer cells are in part inconclusive or even contradictory. While Honjo et al. report loss of (serum-independent) proliferative capacity and abrogation of anchorage-independent growth in breast cancer cells following silencing of Gal-3 expression [26], Matarrese et al. did not observe any difference in cell growth or proliferation upon modulation of Gal-3 expression, but describe enhanced resistance to apoptosis following overexpression of Gal-3 [36]. Likewise, the same group that observed the growth inhibitory effects of Gal-3 silencing in breast cancer cells described strong anti-tumor effects of Gal-3 knockdown in prostate cancer cells, including reduced cell migration, invasion, cell proliferation, anchorage-independent colony formation, and tumor growth in nude mouse xenografts [24]. In contrast, Califice et al. did not observe any effect of Gal-3 expression on proliferation of prostate cancer cells in any constellation, and reported that Matrigel invasion, anchorage-independent growth and nude mouse xenograft formation was promoted only by cytoplasmic Gal-3 expression, while nuclear localization of Gal-3 had the opposite effect [37].

A common theme in many of these studies is the fact that often very few or only a single cell line was analyzed, and that some of the effects were only observed under very specific experimental conditions. We therefore decided to conduct a comprehensive study on the putative role of Gal-3 in pancreatic cancer cells by analyzing in parallel the effect of transient silencing of Gal-3 in a panel of five well-established cell lines with high endogenous Gal-3 expression, as well as study the long-term effects of modulation of Gal-3 expression by stable knockdown in S2-007 cells (high endogenous Gal-3 expression) or stable overexpression in PaTu 8988t cells (negligible endogenous Gal-3 expression), respectively.

Our results demonstrate that transient knockdown of Gal-3 expression resulted in moderate inhibitory effects on proliferation, migration or anchorage-independent growth in individual pancreatic cancer cell lines. These isolated effects were reminiscent of some of the results described in other *in vitro* cancer cell systems. However, although the five cell lines expressed endogeneous Gal-3 at very comparable levels, both on the mRNA as well as on the protein level, functional effects remained limited to individual cell lines and failed to show a consistent pattern across the spectrum of analyzed cell lines. Subcellular localization of the protein did not vary systematically and did not provide an explanation for the differences in susceptibility to Gal-3 knockdown, contrary to the suggestion by Califice et al. [37].

Moreover, functional effects of the modulation of Gal-3 expression were not observed in stable knockdown or overexpression approaches *in vitro*. Most importantly, the attenuation of anchorage-independent growth of S2-007 cells after transient Gal-3 knockdown did not translate into significant differences in growth characteristics of stable knockdown and control clones in nude mouse xenograft tumors *in vivo*.

While this manuscript was in preparation, a study by Kobayashi et al. was published that examined the effects of transient Gal-3 silencing in Panc-1, AsPC-1 and BxPc-3 pancreatic cancer cell lines [39]. Similar to our results, the authors observed no influence on proliferation of the cells, but contrary to our results describe an inhibitory effect of Gal-3 knockdown on cell migration and invasion. While the observed effects were consistent across the three cell lines, only one siRNA sequence was used in the study, and no *in vivo* experiments were performed. It is interesting to note that in our experiments, we observed significant inhibition of cell migration in S2-007 and MIA PaCa-2 cells with one of the two siRNAs used (siGal-3 1); however, this effect was not observed for siGal-3 2, and was altogether absent in the other cell lines (Fig. 6). From the data presented by Kobayashi et al., it can not be ruled out that the observed effects were either specific to the three cell lines used and/or were attributable to off-target effects of the single siRNA used in the study.

Our results do not rule out paracrine and/or systemic effects of the expression of (secreted) Gal-3 by pancreatic cancer cells. Jiang et al. have recently suggested that Gal-3 plays a role in the interaction between SW1990 pancreatic cancer cells and specialized stromal cells of the pancreas termed pancreatic stellate cells (PSC) [40]. In another very recent study, Senapati et al. described that binding of exogenous Gal-3 to MUC4 on the cell surface of CD18/HPAF pancreatic cancer cells enhanced the attachment of the cancer cells to HUVEC endothelial cells in *in vitro* assays, speculating that MUC4/Gal-3 interaction may facilitate extravasation and metastasis of tumor cells *in vivo* [41]. However, the clinical significance of these observations remains unclear, especially in view of the fact that no Gal-3 secretion by CD18/HPAF cells was detected. Moreover, in a large study with 104 pancreatic cancer cases, Shimamura et al. reported a statistically highly significant correlation of high expression of Gal-3 in the primary tumors with reduced metastatic potential and increased post-operative survival [42].

In summary, experimental data on the putative pathophysiological role(s) of Gal-3 in cancer remain controversial, and a clear picture of the function of Gal-3 in the onset and progression of cancer in general as well as pancreatic cancer in particular is not yet emerging.
